# Reframing healthcare violence as systemic failure

**DOI:** 10.1002/jhm.70276

**Published:** 2026-02-08

**Authors:** Minal R. Patel, Patrick M. Carter, Marc A. Zimmerman

**Affiliations:** ^1^ School of Public Health University of Michigan Ann Arbor Michigan USA; ^2^ Institute for Firearm Injury Prevention University of Michigan Ann Arbor Michigan USA; ^3^ Michigan Medicine Department of Emergency Medicine University of Michigan Ann Arbor USA; ^4^ Department of Emergency Medicine Hurley Medical Center Flint Michigan USA

## Abstract

Healthcare workers face escalating violence despite significant security investments, suggesting current approaches miss fundamental causes. We argue that most healthcare violence stems not from individual pathology but from systemic failures—financial barriers, insurance denials, access delays, and administrative complexity—that create volatile patient–provider interactions. Healthcare workers become targets for anger about institutional dysfunctions they cannot control. Current prevention strategies emphasizing individual risk assessment and physical security fail to address these root causes. We propose a research agenda examining connections between system failures and violence, potentially identifying upstream interventions that complement existing security measures while targeting the healthcare dysfunctions driving this epidemic.

## Visual Abstract

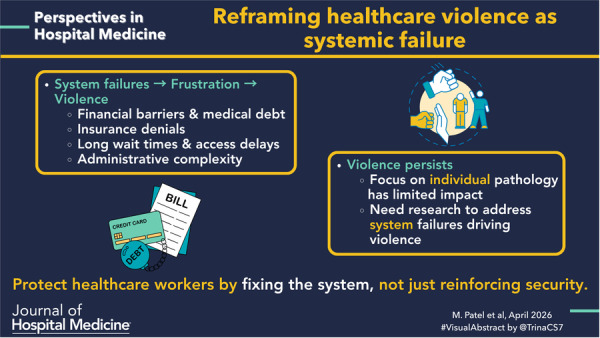

## INTRODUCTION

Healthcare workers across the United States face an escalating epidemic of violence. They are five times more likely to be assaulted than other US workers and account for nearly three‐quarters of all nonfatal workplace violence injuries.^1^ Emergency departments report the highest rates, with 76% of nurses experiencing workplace violence.^2^ This crisis leads to post‐traumatic stress distorder, burnout, staff turnover, and compromised patient care^3^, ^4^, ^5^, ^6^, ^7^ consequences that ripple throughout the healthcare system. These dynamics unfold against broader societal increases in incivility and gun violence fears.^8^

Despite widespread recognition and significant investments in security measures,^3^ healthcare violence continues to escalate. We argue this persistence reflects a fundamental misunderstanding of the problem's origins.

## A SYSTEMIC PERSPECTIVE ON HEALTHCARE VIOLENCE

Most healthcare violence does not stem from psychiatric pathology or criminal intent^9^ we propose that it emerges from the collision between vulnerable patients and a dysfunctional healthcare system. Healthcare workers inadvertently become institutional representatives upon whom patient anger about system failures gets projected, despite lacking the authority to address underlying problems.

Four key system failures create particularly volatile conditions for patient–provider interactions:

*Financial barriers and medical debt* may represent the most significant violence trigger. The US healthcare system remains prohibitively expensive,^10^ creating financial distress that patients direct toward healthcare workers who become the visible face of financial harm despite lacking authority over billing policies or treatment costs.

*Insurance denials* create situations where patients feel abandoned while providers face pressure to deliver care within coverage constraints. For example, policies like emergency medical treatment and active labor act that mandate emergency care while balance billing restrictions simultaneously limit reimbursement, creates financial uncertainty that patients may blame on providers.^11^ Coverage exclusions and claim denials generate frustration that staff must navigate without the power to change decisions.

*Access barriers* including extended wait times generate stress that can escalate to violence. Emergency department experience suggests correlations between wait times and aggressive incidents.^3^, ^6^, ^12^ The nationwide ED boarding crisis exemplifies how capacity constraints create volatile conditions.^13^ Admitted patients languish in emergency departments for days due to bed shortages, experiencing extended waits in chaotic environments not designed for prolonged stays. This structural problem—driven by staffing shortages, insurance authorization delays, and post‐acute care capacity gaps—concentrates multiple system failures in a single high‐stress setting where healthcare workers become targets for accumulated frustration.

*Administrative complexity* breeds institutional distrust directed at frontline workers who must explain systems they did not design. Healthcare billing involves multiple entities, creating confusion about financial responsibility that staff must clarify while lacking the authority to expedite approvals or resolve systemic problems.

These systemic stressors interact with individual factors including coping skills, social support, and mental health status, with patients possessing strong protective factors better able to navigate system failures without resorting to aggression. However, these systemic failures disproportionately impact patients from marginalized communities—while those with financial resources can bypass barriers through very important person services and concierge arrangements, patients on Medicaid or inadequate private insurance face persistent denials and access restrictions. Moreover, reactive security measures like behavioral contracts and electronic health record alerts are inequitably applied, disproportionately targeting Black patients,^14^ compounding historical mistrust and potentially escalating rather than preventing violence. This pattern suggests that current approaches may inadvertently perpetuate systemic inequalities while failing to address root causes affecting the most vulnerable populations.

## WHY CURRENT APPROACHES MISS THE MARK

Healthcare violence prevention has traditionally focused on identifying dangerous individuals and implementing physical security measures.^3^ Current models emphasize psychiatric disorders, substance use, and behavioral warning signs to flag high‐risk patients.^15^ Healthcare facilities invest heavily in panic buttons, security guards, controlled entry points, and threat assessment protocols, including active shooter protocols and metal detectors responding to broader fears of gun violence in public spaces.^16^ These defensive strategies mirror approaches in school safety that emphasize surveillance^17^ rather than addressing underlying causes. While providing some immediate protection, this reactive framework has failed to reduce overall healthcare violence despite significant security investments.

The individual risk model fails to explain why healthcare settings experience disproportionate violence compared with other service industries.^3^ If violence were primarily about individual pathology, we would expect similar rates across all customer service environments. The healthcare‐specific epidemic suggests that something about the healthcare system itself contributes to aggressive incidents.

By focusing exclusively on individual factors, current approaches miss the systemic triggers that create volatile conditions. This narrow focus limits prevention strategies to reactive measures rather than addressing root causes.

## EVIDENCE SUPPORTING SYSTEMIC CONTRIBUTIONS

Healthcare workers often report anecdotally that aggressive incidents coincide with billing discussions, insurance denial notifications, or lengthy wait periods, yet these systemic factors remain absent from most violence prevention frameworks. Case studies of healthcare facility violence often reveal histories of perceived mistreatment, unmet care expectations, or financial grievances.^3^

Healthcare worker violence is a global phenomenon, yet reliable cross‐national data on prevalence and contributing factors remain limited.^18^ Preliminary observations suggest countries with universal healthcare, transparent pricing, and efficient administrative systems may see fewer incidents of patient‐perpetrated violence, though rigorous comparative studies are lacking.^19^

While multiple factors influence these differences, reduced financial stress and simplified administrative processes may protect against healthcare workplace violence.

## A RESEARCH AGENDA FOR UNDERSTANDING SYSTEMIC TRIGGERS

Rigorous empirical work is needed to document and quantify connections between system failures and healthcare violence. Critical research questions include: Which specific system dysfunctions most strongly predict violent incidents? How do financial stressors, insurance denials, access delays, and administrative complexity interact to escalate patient frustration? What organizational policies inadvertently contribute to violence risk?

Prospective studies could track relationships between insurance denials, billing disputes, access delays, and subsequent aggressive events. Healthcare organizations could serve as natural laboratories for comparing violence rates before and after implementing systemic improvements such as transparent billing practices, financial counseling programs, and patient navigation services.

Cross‐national comparative studies could identify which structural features of healthcare systems best protect against workplace violence. Research should also examine whether structural interventions prove more cost‐effective than individual‐focused approaches, potentially offering upstream solutions that address root causes rather than just managing consequences.

Understanding these pathways could inform evidence‐based interventions that complement existing security measures while targeting the system dysfunctions that may be driving much of the violence healthcare workers face daily.

## CONCLUSION

Violence in healthcare settings deserves a more nuanced understanding than current paradigms offer. While individual and clinical risk factors remain important, the potential role of systemic failures—financial barriers, administrative complexity, and access hurdles—warrants serious investigation.

This systemic perspective opens new avenues for research and may identify previously overlooked opportunities for violence prevention. By exploring connections between healthcare system dysfunctions and workplace violence, we can develop interventions that complement existing security measures with upstream approaches targeting root causes.

Research examining these relationships could enhance our understanding while better protecting those who provide care. This reframing invites us to consider not just how to respond to violence, but how healthcare institutions might unintentionally create conditions where such incidents become more likely.

## CONFLICT OF INTEREST STATEMENT

The authors declare no conflicts of interest.
